# The short chain fatty acid propionate stimulates GLP-1 and PYY secretion *via* free fatty acid receptor 2 in rodents

**DOI:** 10.1038/ijo.2014.153

**Published:** 2014-09-09

**Authors:** A Psichas, M L Sleeth, K G Murphy, L Brooks, G A Bewick, A C Hanyaloglu, M A Ghatei, S R Bloom, G Frost

**Affiliations:** 1 Nutrition and Dietetic Research Group, Section of Investigative Medicine, Division of Diabetes, Endocrinology and Metabolism, Department of Medicine, Imperial College, London, UK; 2 Section of Investigative Medicine, Division of Diabetes, Endocrinology and Metabolism, Department of Medicine, Imperial College, London, UK; 3 Division of Diabetes & Nutritional Sciences, Kings College London, Yeovil, UK; 4 Department of Surgery and Cancer, Institute of Reproductive and Developmental Biology, Imperial College, London, UK

## Abstract

**Background and Objectives:**

The gut hormones peptide YY (PYY) and glucagon-like peptide 1 (GLP-1) acutely suppress appetite. The short chain fatty acid (SCFA) receptor, free fatty acid receptor 2 (FFA2) is present on colonic enteroendocrine L cells, and a role has been suggested for SCFAs in appetite regulation. Here, we characterise the *in vitro* and *in vivo* effects of colonic propionate on PYY and GLP-1 release in rodents, and investigate the role of FFA2 in mediating these effects using FFA2 knockout mice.

**Methods:**

We used Wistar rats, C57BL6 mice and free fatty acid receptor 2 knockout (FFA^−/−^) mice on a C57BL6 background to explore the impact of the SCFA propionate on PYY and GLP-1 release. Isolated colonic crypt cultures were used to assess the effects of propionate on gut hormone release *in vitro*. We subsequently developed an *in vivo* technique to assess gut hormone release into the portal vein following colonic infusion of propionate.

**Results:**

Propionate stimulated the secretion of both PYY and GLP-1 from wild-type primary murine colonic crypt cultures. This effect was significantly attenuated in cultures from FFA2^−/−^ mice. Intra-colonic infusion of propionate elevated PYY and GLP-1 levels in jugular vein plasma in rats and in portal vein plasma in both rats and mice. However, propionate did not significantly stimulate gut hormone release in FFA2^−/−^ mice.

**Conclusions:**

Intra-colonic administration of propionate stimulates the concurrent release of both GLP-1 and PYY in rats and mice. These data demonstrate that FFA2 deficiency impairs SCFA-induced gut hormone secretion both *in vitro* and *in vivo*.

## Introduction

The anorexigenic gut hormone peptide YY (PYY) and the incretin glucagon-like peptide 1 (GLP-1) acutely suppress appetite following their co-release from enteroendocrine L cells.^1^ Evidence suggests that the displacement of nutrients in the gut caused by procedures such as the RYGB (roux-en-Y gastric bypass) may mediate the dramatic effects on weight loss and type 2 diabetes of these surgeries, at least in part through the release of PYY and GLP-1.^2,3^ An understanding of how nutrients stimulate PYY and GLP-1 secretion could lead to more effective nutritional management and/or prevention of obesity.

Nutrient-stimulated gut hormone release from L cells is well documented.^4^ However, the majority of ingested nutrients are absorbed in the small intestine, before reaching the large intestine which harbours the highest density of L cells.^5^ Interestingly, the colon is a major site of gut bacterial fermentation, yielding high levels of short chain fatty acids (SCFAs, 70–130 mmol l^−1^).^6^ The main substrates for the production of SCFAs by the colonic microbiota are dietary carbohydrates that have escaped digestion in the small intestine, collectively referred to as dietary fibre. It is of interest that over man's evolution the amount of plant material consumed has decreased tremendously; the daily dietary fibre intake in hominins (who mainly consumed grasses and sedges) is estimated to have been over 100 g, while the modern western diet often results in daily intake below 15 g. Therefore, the amount of material being fermented in the colon has reduced markedly.

SCFAs mediate some of their biological effects *via* the G protein-coupled receptors FFA2 and FFA3, and there has been increased interest recently in the beneficial role of FFA2 in gastrointestinal physiology and immune function, as well as in energy and glucose homeostasis.^7, 8, 9, 10^ It was recently demonstrated that FFA2 protects mice from hyperphagia, obesity and insulin resistance on a high-fat diet.^7^ Furthermore, FFA2-deficient mice have been reported to demonstrate impaired glucose tolerance on a normal chow diet.^10^ These effects may be accounted for, at least in part, by SCFA- and FFA2-mediated stimulation of gut hormone release. Indeed, Tolhurst *et al.*^10^ recently demonstrated that active GLP-1 secretion in response to SCFAs is attenuated from primary FFA2^−/−^ murine L cells *in vitro*. However, the effect of SCFAs on PYY secretion from primary colonic cultures has not previously been investigated, and it is unknown whether FFA2-dependent effects on gut hormone release *in vitro* are relevant *in vivo*. Recent evidence suggests that the SCFA propionate, which has a high affinity for FFA2,^11^ may have a role in the enhanced gut hormone release observed following RYGB for the treatment of obesity.^12^ In addition, propionate is an end-product of fermentation and thus is not cross-metabolised by the microbiota unlike acetate and butyrate.^13^

We therefore aimed to characterise in rodents (1) the effect of colonic propionate on PYY and GLP-1 release, and (2) the role of FFA2 in mediating these effects, both *in vitro* and *in vivo*.

## Materials and methods

### *In vitro*

#### Colonic culture preparation

The colons of male C57BL6 mice (⩾8 weeks of age; Harlan Laboratories, Bicester, UK) were removed, cleaned and placed into ice-cold L-15 (Leibowitz) medium (PAA, Yeovil, UK). The intestinal tissue was thoroughly cleaned with L-15 medium and digested with 0.4 mg ml^−1^ collagenase XI (Sigma, Poole, UK) in high-glucose DMEM at 37 ^o^C, as described previously.^14^ Resulting cell suspensions were centrifuged (5 min, 300 *g*) and the pellets were resuspended in DMEM (supplemented with 10% fetal calf serum and 1% antibiotics, 100 U ml^−1^ penicillin and 0.1 mg ml^−1^ streptomycin). Combined cell suspensions were filtered through a nylon mesh (pore size ~250 μm) and plated onto 24-well, 1% Matrigel-coated plates. The plates were incubated overnight at 37 ^o^C in an atmosphere of 95% O_2_ and 5% CO_2_.

#### Gut hormone secretion experiments

Secretion experiments were carried out within 24 h of plating. The cells were washed three times with secretion buffer (4.5 mM KCl, 138 mM NaCl, 4.2 mM NaHCO_3_, 1.2 mM NaH_2_PO_4_, 2.6 mM CaCl_2_, 1.2 mM MgCl_2_, and 10 mM HEPES, which was adjusted to pH 7.4 with NaOH) supplemented with 0.1% fatty acid-free bovine albumin serum (BSA; Sigma). The cells were then incubated in secretion buffer containing test reagents for 2 h at 37 ^o^C in an atmosphere of 95% O_2_ and 5% CO_2_. The adenylyl cyclase activator forskolin (Sigma) and the phosphodiesterase inhibitor 3-isobutyl-1-methylxanthine (IBMX; Sigma) were prepared as 10 mmol l^−1^ stock solutions in dimethyl sulfoxide and used at a final concentration of 10 μmol l^−1^ each. Test solutions were prepared on the day of the secretion experiment. Test reagents were not cytotoxic as determined by a lactate dehydrogenase cytotoxicity assay (G-Biosciences, Maryland Heights, MO, USA).

Following incubation, cell supernatants were collected and centrifuged (3 min, 100 *g*). The resulting supernatants were then stored at −20 ^o^C pending analysis. The plated cells were treated with cell lysis buffer and scraped, and following centrifugation the lysates were stored at −20 ^o^C pending analysis.

Gut hormone secretion was calculated as a fraction of the total hormone (secreted+extracted) measured from each well and expressed relative to basal secretion measured during the same experiment.

### *In vivo*

#### Animals and housing

All animal procedures undertaken were approved by the local ethics committee and conformed to Home Office regulations. On arrival, male Wistar rats (Charles River, Margate, UK) or male C57BL6 mice (Harlan Laboratories) were housed in pairs and maintained at 21–23 ^o^C on a 12-h light, 12-h dark cycle (light period 0700–1900 h). During the 72-h acclimatisation period, all rodents were given *ad libitum* access to water and RM1 standard chow (RM1 diet; Special Diet Services Ltd., Witham, Essex, UK). FFA2 knockout mice were obtained from Professor McKay at the Garven Institute. FFA2 was knocked out by homologous recombination which substitutes 55 bp of FFA2 exon 1 with the β-gal-neo cassette, shifting the downstream amino-acid sequence out of the reading frame.^15^

#### Intra-colonic administration of propionate in rats

Male Wistar rats (200–250 g) were fasted overnight (with *ad libitum* access to water) and anaesthetised under isoflurane (1.5–4% 2 L per minute O_2_ flow). A jugular vein cannulation and laparatomy were performed. Two baseline jugular vein blood samples were collected at *t*=−15 and *t*=0 min. Propionic acid (180 mmol l^−1^, 2.5 ml (~0.45 mmol) pH 5.5 using NaOH) or saline control (matched for pH and sodium content) was injected into the rat proximal colon distal to the caecum. At *t*=15 min a portal vein sample and a jugular vein sample were collected. Further jugular vein samples were collected at *t*=30 and *t*=60 min. Blood samples were collected into eppendorfs containing DPPIV inhibitor (Millipore, Abingdon, Oxfordshire, UK; 1 μl per 100 μl blood, 100 μmol l^−1^ final concentration) and protease inhibitor cocktail (Sigma; 1 μl per 100 μl blood). Blood samples were centrifuged for 10 min for separation of plasma. Separated plasma was placed immediately on dry ice. Samples were stored at −20 ^o^C pending gut hormone analysis.

#### Intra-colonic administration of propionate in wild-type and FFA2^−/−^ mice

Male C57BL6 or FFA2^−/−^ mice were anaesthetised under isoflurane (1.5–4% 2 L per minute O_2_ flow) and a laparotomy was performed. The mice received an intra-colonic injection (300 μl) of saline or propionic acid (180 mmol l^−1^, matched for pH and sodium content). Portal vein blood was collected 5 min post injection, using an established sampling technique.^16,17^ Plasma was separated and stored as above.

#### Gut hormone analysis

Total GLP-1 and PYY levels in cell supernatants and lysates, and in plasma, were measured using sensitive and specific in-house radioimmunoassays as previously described.^18,19^

#### Statistical analysis

Normality was determined using the D'Agostino-Pearson omnibus test where *n*⩾8 per group. Statistical significance was calculated by unpaired *t*-test, one-way ANOVA or two-way ANOVA, as appropriate. Pairwise comparisons were carried out using a Bonferroni multiple comparison *post hoc* test. Statistical significance was accepted at *P*<0.05 throughout. Data are presented as mean±s.e.m. Analysis was carried out using Graph Pad Prism software, version 5.0 (La Jolla, CA, USA).

## Results

### Propionate stimulates the release of PYY and GLP-1 from primary murine L cells

**Figure 1:**
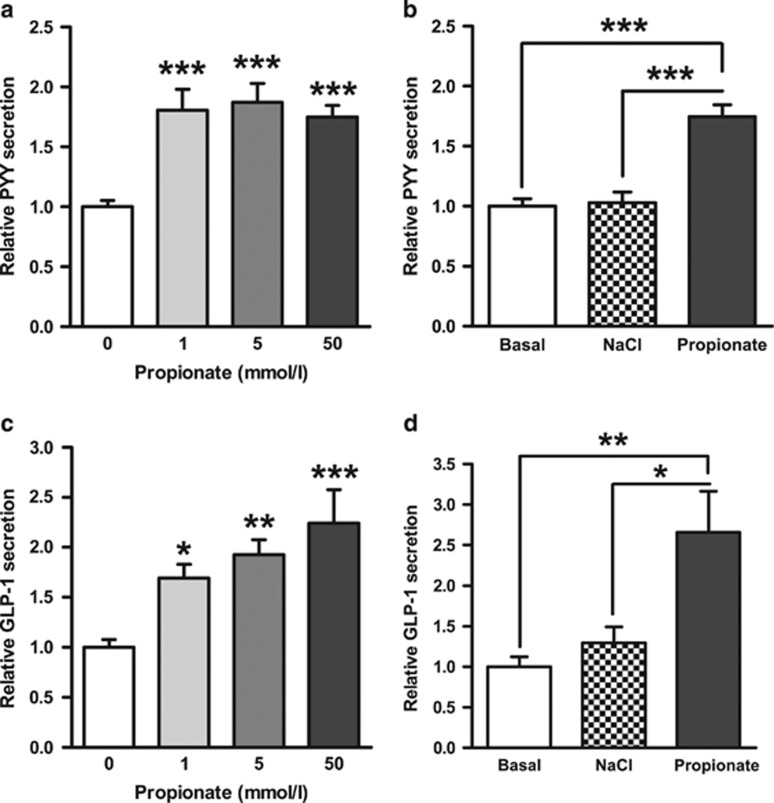
The effect of propionate on GLP-1 and PYY secretion from primary murine L cells. Mixed primary colonic cultures were incubated with propionate (1–50 mmol l^−1^) (**a**, **c**). At a concentration of 50 mmol l^−1^, NaCl had no effect on gut hormone release (**b**, **d**). GLP-1 and PYY secretion in each well was expressed as a percentage of total GLP-1 or PYY contained within the well and normalised to the basal secretion measured in parallel within the same experiment. Data represent means±s.e.m. (*n*=6–24 wells). Significance is shown relative to basal secretion (0 mmol l^−1^) (**a**, **c**) or to the iso-osmotic NaCl control (**b**, **d**) using one-way ANOVA (**a**, *F*=11.73, *P*<0.0001; **b**, *F*=7.931, *P*=0.0022; **c**, *F*=24.75, *P*<0.0001; **d**, *F*=25.99, *P*<0.0001) with a Bonferroni *post hoc* test (*P<0.05, **P<0.01, ***P<0.001).

In primary murine colonic cultures, physiological concentrations of propionate (1–50 mmol l^−1^)^20^ significantly stimulated GLP-1 and PYY secretion over a 2-h incubation (Figures 1a and c). The higher concentration of propionate induced a 1.8- and 2.2-fold increase in PYY and GLP-1 release, respectively. Furthermore, the effect of 50 mmol l^−1^ propionate on gut hormone release remained highly significant when compared with an iso-osmotic NaCl control (Figures 1b and d).

### Intra-colonic administration of propionate increases circulating and portal vein plasma PYY and GLP-1 concentrations in rats

**Figure 2:**
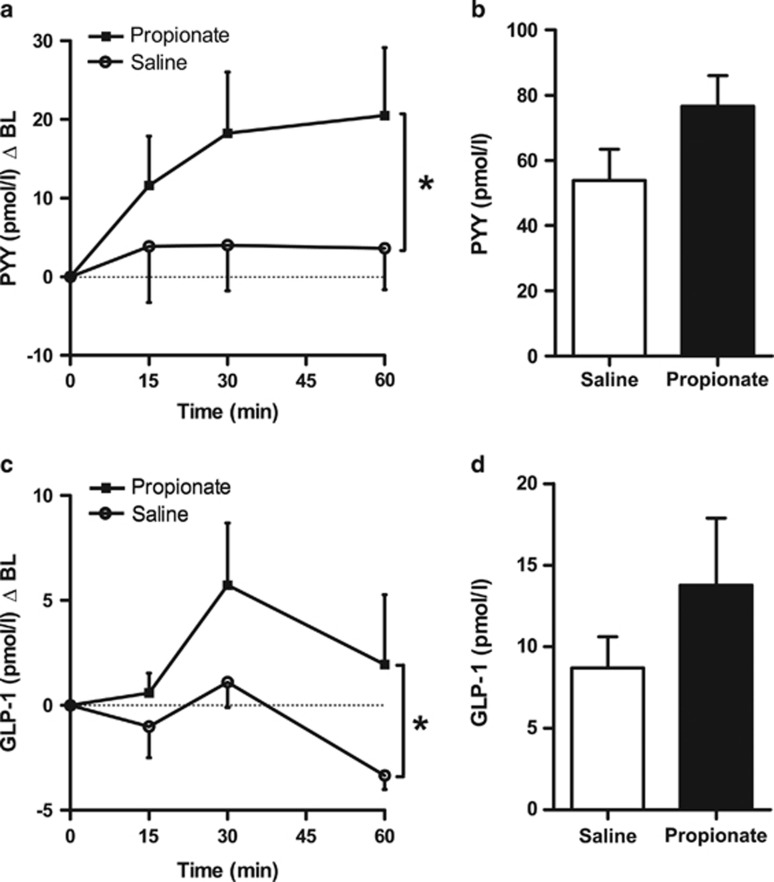
Intra-colonic administration of propionate increases jugular and portal vein plasma gut hormone concentrations in male Wistar rats. Blood samples were collected over a 60-min period, *via* a jugular vein cannula (**a**, **c**), and at *t*=15 min from the portal vein (**b**, **d**), following an intra-colonic injection (2.5 ml) of 180 mmol l^−1^ propionate or saline (matched for pH and sodium content) in isoflurane-anaesthetised rats. Data represent means±s.e.m. (*n*=10–14 per group). Significance was determined using two-way ANOVA (**a**, **c**) or unpaired *t*-test (**b**, **d**) (*P<0.05).

An *in vivo* model was developed to enable the assessment of plasma gut hormone profiles, induced by a single intra-colonic injection of propionate. Propionate was injected into the colon, jugular vein blood samples collected over the following 60-min time period, and a single blood sample taken from the portal vein at 15 min. An intra-colonic injection of 180 mmol l^−1^ propionate (a total dose of ~0.45 mmol) vs saline control (matched for sodium content and pH) in anaesthetised rats resulted in a significant rise in circulating plasma PYY and GLP-1 levels (two-way ANOVA, effect of treatment *P*=0.024 and *P*=0.023, respectively). Circulating plasma GLP-1 levels peaked at 30 min, whereas plasma PYY levels rose steadily and remained elevated at 60 min (Figures 2a and c). Furthermore, portal vein PYY and GLP-1 levels at 15 min were elevated compared with saline (PYY, 76.5±9.4 vs 53.8±9.6 pmol l^−1^ and GLP-1, 13.8±4.1 vs 8.7±1.9 pmol l^−1^), but these differences were not statistically significant (Figures 2b and d).

### Propionate-induced gut hormone release is attenuated in FFA2^−/−^ primary murine L cells

**Figure 3:**
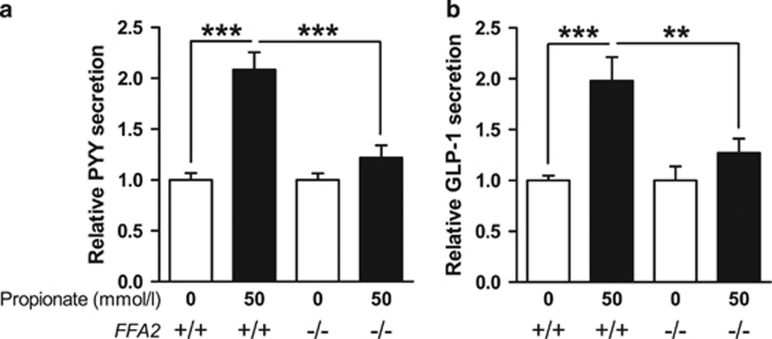
Propionate-induced PYY and GLP-1 secretion is attenuated in FFA2^−/−^ primary murine L cells. Primary colonic cultures from FFA2 knockout (−/−) or WT (+/+) littermates were incubated with or without propionate (50 mmol l^−1^). PYY (**a**) and GLP-1 (**b**) secretion in each well was expressed as a percentage of total PYY or GLP-1 contained within the well and normalised to the basal secretion (0 mmol l^−1^) measured in parallel within the same experiment. Data represent means±s.e.m. (*n*=22–38 wells). Significance is shown relative to basal secretion using one-way ANOVA (**a**, *F*=19.31, *P*<0.0001; **b**, *F*=8.816, *P*<0.0001) with a Bonferroni *post hoc* test (**P<0.01; ***P<0.001).

To evaluate the role of FFA2 in mediating propionate-induced PYY and GLP-1 secretion, we examined the effect of propionate on gut hormone secretion from primary colonic cultures from FFA2-deficient mice (FFA2^−/−^). Incubation of wild-type (WT) murine colonic cultures with 50 mmol l^−1^ propionate robustly stimulated PYY and GLP-1 secretion from primary L cells (~2-fold, Figures 3a and b). However, the response to propionate (relative to basal) was markedly attenuated in FFA2^−/−^ colonic cultures compared with WT (PYY, 1.2- vs 2.1-fold and GLP-1, 1.3- vs 2.0-fold) (Figures 3a and b).

### Intra-colonic propionate increases plasma gut hormone levels *via* an FFA2-dependent mechanism in mice

**Figure 4:**
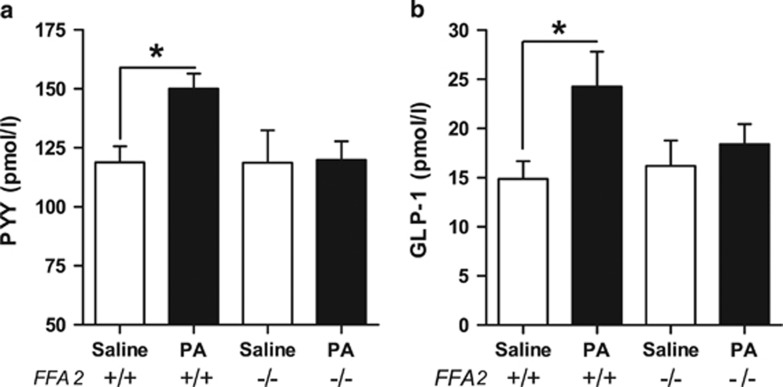
Intra-colonic administration of propionate increases portal vein plasma PYY and GLP-1 levels *in vivo* in mice *via* an FFA2-dependent mechanism. Blood samples were collected from the portal vein 5 min after an intra-colonic injection (300 μl) of propionate (180 mmol l^−1^) or saline (matched for pH and sodium content) in isoflurane-anaesthetised FFA2 knockout (−/−) mice or WT littermates (+/+). Data represent means±s.e.m. (*n*=5–7 per group). Significance was determined using one-way ANOVA (**a**, *F*=2.457, *P*=0.094; **b**, *F*=2.660, *P*=0.076) with a Bonferroni *post hoc* test (*P<0.05).

To determine whether the reduced PYY and GLP-1 secretory responses to propionate observed in primary colonic cultures from FFA2^−/−^ mice *in vitro* translated into impaired gut hormone secretion from these mice *in vivo*, portal vein plasma PYY and GLP-1 responses to a single intra-colonic injection of propionic acid (180 mmol l^−1^) or saline control were investigated in FFA2^−/−^ mice compared with their WT littermates. In the WT animals, propionate led to a significant 1.3- and 1.6-fold increase in portal vein PYY and GLP-1 levels above saline, respectively (both *P*<0.05) (Figure 4). No change was observed in the FFA2^−/−^ group. There was no difference between the two genotypes in portal vein plasma GLP-1 and PYY levels 5 min after an intra-colonic administration of saline (Figure 4).

## Discussion

Our studies demonstrate that intra-colonic administration of propionate stimulates the concurrent release of both GLP-1 and PYY in rodents, and demonstrate *in vitro*, and for the first time *in vivo*, that FFA2 deficiency impairs SCFA-induced gut hormone secretion.

It has been hypothesised for some time that SCFAs acting *via* their receptors FFA2 and FFA3, which are enriched in colonic enteroendocrine L cells, stimulate the release of anorexigenic and incretin gut hormones.^10,21,22^ In support of this hypothesis, recent work by Tolhurst *et al.*^10^ demonstrated that FFA2^−/−^ primary colonic cultures have an attenuated GLP-1 response to SCFAs. Our work confirms this effect, and also demonstrates that SCFA-stimulated PYY release is attenuated in the same model.

Even at the low concentration of 1 mmol l^−1^, propionate was able to significantly induce both GLP-1 and PYY release from murine primary L cells. These results are in accordance with previous findings for GLP-1.^8,10^ In contrast to the high physiological concentrations of SCFAs reported in the gut lumen, 1 mmol l^−1^ is more in line with the EC_50_ of FFA2 for SCFAs.^11,23^ Several hypotheses have been put forward to account for this discrepancy. Firstly, it is possible that L cells *in vivo* are exposed to lower SCFA concentrations due to absorption of SCFAs by surrounding colonocytes and/or due to the presence of the mucous layer.^10^ Therefore, the luminal concentration may not reflect the concentration at the level of the L cell surface. Secondly, Nøhr *et al.^8^* proposed that colonic enteroendocrine cells may sense the considerably lower concentration of SCFAs found at the basolateral surface.^8^ Alternatively, Tolhurst *et al.*^10^ also speculated that colonic SCFAs may have a role in providing a chronic stimulatory tone on L cells *via* apical or basolateral SCFA receptors, which could account for the presence of circulating gut hormones in the fasted state. In our studies, we were unable to detect a difference in fasting levels of GLP-1 (following saline injection), but this may be due to a difference in fasting duration (4 h vs overnight). The longer fasting period would be expected to reduce colonic SCFA levels and thus reduce stimulatory tone at the receptor.

It is critical to demonstrate that findings *in vitro* also translate into the *in vivo* setting. In this context, it was important to demonstrate that luminal propionate was able to stimulate gut hormone release. Furthermore, in light of the differential release of GLP-1 and PYY observed under certain conditions in response to SCFAs in rats,^24,25^ measurement of both gut hormones in parallel was necessary. Intra-colonic administration of propionate significantly increased plasma levels of both gut hormones in rats and mice.

A model was developed to enable the simultaneous measurement of gut hormone levels in both the portal and peripheral circulation following intra-colonic administration of propionate in anaesthetised rats. The observed 18.6- and 20.9 pmol/l rise in circulating plasma PYY levels at 30 and 60 min is in line with previous studies which administered a mixture of SCFAs.^26,27^ In contrast to previous studies that failed to show an effect of SCFAs on GLP-1,^24,25^ the data presented here suggest that both PYY and GLP-1 were elevated in parallel following intra-colonic administration of propionate. However, the GLP-1 response was more transient in nature; GLP-1 peaked at 30 min but the levels were not maintained and were reduced at 60 min.

Portal vein GLP-1 concentrations recorded in this study (8.7±1.9 pmol l^−1^) were similar to those reported in the literature (7.8±0.7 pmol l^−1^ (ref. 16) and 9.0±0.7 pmol l^−1^ (ref. 28)). Portal vein plasma levels of both PYY and GLP-1 were elevated 15 min following the administration of propionate compared with saline (1.4- and 1.6-fold, respectively), though these differences were not statistically significant. However, it is possible that the time point chosen was too delayed to detect the peak in portal vein gut hormone levels; significantly increased portal vein gut hormone levels were detected at 5 min in the mouse study.

In this paper, we have chosen to focus on the SCFA propionate. Propionate-induced PYY and GLP-1 release was significantly lower from primary colonic cultures derived from FFA2^−/−^ mice compared with WT cultures. However, the FFA2^−/−^ colonic cultures maintained a robust gut hormone response to elevated intracellular cAMP concentrations (Supplementary Figure 1), suggesting that the intracellular machinery required for gut hormone release is intact.

Despite the fact that the majority of *in vitro* work has been carried out in primary murine L cells and mouse-derived cell lines, the effect of colonic administration of SCFAs on plasma gut hormone levels in mice has not previously been investigated. The rat portal vein sampling procedure described above was adapted for use in mice, to enable the measurement of portal vein plasma gut hormone levels following intra-colonic administration of propionate. Basal GLP-1 values (13.1±8.5 pmol l^−1^) were similar to those reported in the literature (~16 pmol l^−1^, ref. 17). Notably, both basal and stimulated mouse portal vein gut hormone levels were higher than those in rats (basal PYY, ~2-fold and GLP-1, ~1.7-fold). Nevertheless, our results demonstrate that colonic propionate increases the portal vein levels of both PYY and GLP-1 by a similar magnitude in both mice and rats.

Intriguingly, levels of the SCFA propionate, a potent endogenous agonist of FFA2, are elevated following RYGB in rodents.^12,29^ Furthermore, a significant negative correlation between adiposity and caecal butyrate and propionate concentrations has also been reported in germ-free mice receiving faecal transplants from human twin donors discordant for obesity.^30^ While the two published studies that have investigated energy homeostasis in FFA2^−/−^ mice to date led to different conclusions, both reported hyperphagia on an high fat diet compared with WT.^7,31^ In both cases, the high fat diet also contained fibre; 6.5%^7^ and 3.9%.^31^ Our findings suggest that reduced levels of anorexigenic gut hormones may account, at least in part, for this observation.

In our studies, we have used the SCFA propionate to investigate the role of FFA2 activation in PYY and GLP-1 release. A wide range of nutrients are known to stimulate gut hormone secretion from L cells. However, these nutrients do not reach colonic L cells in significant amounts. Therefore, SCFAs are likely to be an important source of colonic L cell stimulation. However, it is also likely that SCFAs take on a more major role when animals are fed a diet high in fermentable fibre. Fermentable fibre and SCFAs have also been demonstrated to increase L cell numbers.^32,33^

We have shown that the SCFA propionate stimulates the release of both GLP-1 and PYY from primary murine colonic cultures and *in vivo* following intra-colonic administration in rodents. The work presented here demonstrates for the first time that propionate-stimulated PYY release from primary FFA2^−/−^ colonic cultures is also significantly attenuated and that, unlike WT animals, FFA2^−/−^ mice do not respond to propionate. Targeting nutrient sensing pathways, such as those activated by SCFAs, may have translational potential by mimicking the elevated gut hormone profiles observed following nutrient displacement procedures thus beneficially modulating appetite.
