# Supplementary material for: The short chain fatty acid propionate stimulates GLP-1 and PYY secretion via free fatty acid receptor 2 in rodents
Source: Int J Obes (Lond). 2014 Sep 9;39(3):424–9. doi: 10.1038/ijo.2014.153 (PMC4356745; doi:10.1038/ijo.2014.153)
Supplement: Supplementary Information [file ijo2014153x1.doc]

**SUPPLEMENTARY FIGURE S1**

Figure S1. *Ffa2^-/-^* primary murine L cells secrete gut hormones in response to an increase in [cAMP]_i_ levels. Primary colonic cultures from *Ffa2* knockout (-/-) or wild type (+/+) littermates were incubated with or without forskolin/IBMX (F/I, 10µmol/l each). PYY (A) and GLP-1 (B) secretion in each well is expressed as a percentage of total PYY or GLP-1 contained within the well and compared to basal secretion measured within the same experiment. Data represent means ± SEM (n=3-7 wells). Significance is shown relative to basal secretion using one-way ANOVA (a, *F*=9.504, P=0.0011; b, *F*=51.64, P<0.0001) with a Bonferroni post hoc test (**P<0.01; ***P<0.001).

**Summary:**

*Ffa2^-^*^/-^ primary L cells maintain a normal response to other stimuli. Both *Ffa2^-/-^* and WT colonic L cells respond to an increase in intracellular cAMP levels by releasing PYY and GLP-1. This suggests that the intracellular machinery required for gut hormone secretion is intact in *Ffa2^-/-^* L cells.
